# Ion channelopathies in human induced pluripotent stem cell derived cardiomyocytes: a dynamic clamp study with virtual I_K1_

**DOI:** 10.3389/fphys.2015.00007

**Published:** 2015-02-03

**Authors:** Rosalie M. E. Meijer van Putten, Isabella Mengarelli, Kaomei Guan, Jan G. Zegers, Antoni C. G. van Ginneken, Arie O. Verkerk, Ronald Wilders

**Affiliations:** ^1^Department of Anatomy, Embryology and Physiology, Academic Medical Center, University of AmsterdamAmsterdam, Netherlands; ^2^Department of Experimental Cardiology, Academic Medical Center, University of AmsterdamAmsterdam, Netherlands; ^3^Department of Cardiology and Pneumology, Georg-August-University of GöttingenGöttingen, Germany

**Keywords:** action potentials, Andersen–Tawil syndrome, cardiac ion channelopathies, inward rectifier potassium channel, KCNJ2 gene, Kir2.1 protein, patch clamp, short QT syndrome 3

## Abstract

Human induced pluripotent stem cell derived cardiomyocytes (hiPSC-CMs) are widely used in studying basic mechanisms of cardiac arrhythmias that are caused by ion channelopathies. Unfortunately, the action potential profile of hiPSC-CMs—and consequently the profile of individual membrane currents active during that action potential—differs substantially from that of native human cardiomyocytes, largely due to almost negligible expression of the inward rectifier potassium current (I_K1_). In the present study, we attempted to “normalize” the action potential profile of our hiPSC-CMs by inserting a voltage dependent *in silico* I_K1_ into our hiPSC-CMs, using the dynamic clamp configuration of the patch clamp technique. Recordings were made from single hiPSC-CMs, using the perforated patch clamp technique at physiological temperature. We assessed three different models of I_K1_, with different degrees of inward rectification, and systematically varied the magnitude of the inserted I_K1_. Also, we modified the inserted I_K1_ in order to assess the effects of loss- and gain-of-function mutations in the *KCNJ2* gene, which encodes the Kir2.1 protein that is primarily responsible for the I_K1_ channel in human ventricle. For our experiments, we selected spontaneously beating hiPSC-CMs, with negligible I_K1_ as demonstrated in separate voltage clamp experiments, which were paced at 1 Hz. Upon addition of *in silico* I_K1_ with a peak outward density of 4–6 pA/pF, these hiPSC-CMs showed a ventricular-like action potential morphology with a stable resting membrane potential near −80 mV and a maximum upstroke velocity >150 V/s (*n* = 9). Proarrhythmic action potential changes were observed upon injection of both loss-of-function and gain-of-function I_K1_, as associated with Andersen–Tawil syndrome type 1 and short QT syndrome type 3, respectively (*n* = 6). We conclude that injection of *in silico* I_K1_ makes the hiPSC-CM a more reliable model for investigating mechanisms underlying cardiac arrhythmias.

## Introduction

Long QT syndrome (LQTS), with an estimated prevalence of 1:2000 (Schwartz et al., 2009), is the most commonly encountered inherited arrhythmia disorder in clinical practice (≈35%), whereas short QT syndrome (SQTS) is the least common one (≈2%) (Hocini et al., 2014). Both can be related to mutations in the *KCNJ2* gene, which encodes the Kir2.1 member of the Kir2 protein family. Tetramers of Kir2 proteins constitute the cardiac inward rectifier potassium channel, which is responsible for maintaining a stable resting membrane potential in the working myocardium by conducting the inward rectifier potassium current (I_K1_) (Dhamoon and Jalife, 2005). I_K1_ is also largely responsible for the final repolarization phase of the ventricular action potential (Shimoni et al., 1992). In human ventricle, Kir2.1 is by far the most abundant Kir2.x protein (92%; Kir2.2: 7%; Kir2.3: <1%). The same holds true for human atrium, but figures are somewhat different (81%; Kir2.2: 9%; Kir2.3: 10%), which may explain the less pronounced rectification of I_K1_ in human atrium (Wang et al., 1998).

A loss-of-function mutation in *KCNJ2* may result in ventricular arrhythmias and abnormalities on the ECG, including prolongation of the QT interval. This explains the classification as LQTS type 7 (LQT7; Tristani-Firouzi et al., 2002). However, the expression of Kir2.1 channels is not limited to the heart and loss-of-function mutations in *KCNJ2* may also lead to periodic paralysis, dysmorphic features and neurocognitive problems. Given the multisystem nature of the disorder associated with suppression of Kir2.1 channel function and the only modest QT prolongation, if any, Andersen–Tawil syndrome (ATS) has become the preferred name (Nguyen et al., 2013). With an estimated prevalence in the order of 1:100,000, ATS is rare. A mutation in *KCNJ2* can be identified in 60–70% of all ATS patients (ATS type 1, ATS1).

Gain-of-function mutations in *KCNJ2* are even more rare. They may cause familial atrial fibrillation type 3 (AF3) as well as SQTS type 3 (SQT3). The increase in I_K1_ results in a shortening of the repolarization phase of the cardiac action potential and a predisposition to supraventricular and ventricular arrhythmias (Patel et al., 2010). Only a few SQT3 mutations have been identified thus far (Priori et al., 2005; Hattori et al., 2012; Deo et al., 2013; Ambrosini et al., 2014). The E299V (Deo et al., 2013) and M301K (Hattori et al., 2012) mutations both lead to a largely abolished rectification of I_K1_, which explains the shortened action potential and QT interval.

Unfortunately, the availability of freshly isolated human cardiomyocytes (CMs) for research is highly limited. This holds for control myocytes and even more so for myocytes from patients with channelopathies. Culturing human CMs or mammalian CMs in general is not an option because these cells lose specific morphologic and functional properties in culture (Mitcheson et al., 1998; Louch et al., 2011). For example, there is a significant decrease in I_K1_ over time.

Functional aspects of LQTS and related monogenic disorders have been investigated by expressing mutant channels in heterologous expression systems like *Xenopus* oocytes, human embryonic kidney (HEK)-293 cells, and Chinese hamster ovary (CHO) cells. However, results from expression studies should be interpreted with care, as illustrated by the essential differences in results obtained from *Xenopus* and HEK-293 expression systems for the 1795insD mutation in *SCN5A*, encoding the pore forming subunit of the fast sodium channel (Remme et al., 2008). Another, highly remarkable illustration comes from the pathological D1275N mutation in *SCN5A*, which produces near-normal currents in multiple heterologous expression experiments (Watanabe et al., 2011).

The generation of transgenic mice allows the study of mutant channel behavior in the native myocyte environment. However, the electrophysiology of murine CMs is essentially different from that in human, with differences in action potential profile and underlying membrane currents and, of course, in heart rate, limiting the usefulness of transgenic mouse models for the research of cardiac ion channelopathies (Nerbonne, 2014).

CMs derived from human induced pluripotent stem cells (hiPSC-CMs), building on the seminal work of Takahashi et al. (2007), are a promising new tool in the research of cardiac ion channelopathies (Zhang et al., 2009; Narsinh et al., 2011). Cell lines can be disease and species dependent, thus not only allowing research on the effects of channelopathies in their natural setting, but also on the role of genetic background. Over the past years, hiPSC-CMs have been used in a growing number of studies on channelopathies, as reviewed by Hoekstra et al. (2012), Priori et al. (2013), Sinnecker et al. (2013), Knollmann (2013), Sallam et al. (2014), Savla et al. (2014), and Shinnawi and Gepstein (2014).

An essential problem with hiPSC-CMs is their immature phenotype. In general, hiPSC-CMs demonstrate spontaneous activity with a depolarized maximum diastolic potential (MDP), a low maximum upstroke velocity [(dV/dt)_max_] and a highly variable action potential duration (Hoekstra et al., 2012). If quiescent, their resting membrane potential (RMP) is depolarized in comparison with that of native working CMs. The depolarized MDP or RMP of hiPSC-CMs results in inactivation and thus a lower functional availability of fast sodium channels, which may explain, at least in part, their low upstroke velocity. Similarly, functional availability of transient outward potassium channels may be reduced through inactivation at depolarized potentials (Cordeiro et al., 2013). Ultrastructurally, there are also differences between hiPSC-CMs and native CMs. In particular, hiPSC-CMs exhibit a poorly developed sarcoplasmic reticulum and a lack of T tubules (Gherghiceanu et al., 2011), which is associated with immaturity of the Ca^2+^ homeostasis (Louch et al., 2015).

A common observation in hiPSC-CMs is their virtual lack of I_K1_ (Hoekstra et al., 2012), even if the age post-differentiation is raised to 120 days (Doss et al., 2012), which readily explains their depolarized MDP or RMP. Several attempts have been proposed to “mature” hiPSC-CMs through an increase in I_K1_. One approach is to mediate the expression of Kir2.1 channels through adenoviral transduction, as Lieu et al. (2013) demonstrated for CMs derived from human embryonic stem cells. Another approach was put forward by Bett et al. (2013). In their pilot study, they increased I_K1_ in hiPSC-CMs through “electronic expression,” using the dynamic clamp technique (Wilders, 2006), and demonstrated that the action potential profile could be improved by eliminating spontaneous activity and establishing a physiological RMP. Of note, their whole cell patch clamp experiments were carried out at room temperature and with an I_K1_ magnitude that was independent of cell size.

In the present study, we increased the expression level of I_K1_ in our hiPSC-CMs by adding *in silico* I_K1_ using a dynamic patch clamp approach. We made perforated patch clamp recordings from a series of hiPSC-CMs at a physiological temperature, systematically varying the magnitude of I_K1_ and assessing the effects of multiple models of I_K1_, with different degrees of rectification. Also, we assessed the effects of both loss-of-function and gain-of-function mutations in I_K1_ by modifying our *in silico* I_K1_, thus simulating ATS1 and SQT3, respectively.

## Materials and methods

### Cell preparation

#### Generation of human induced pluripotent stem cells

Skin punch biopsies were taken from adult healthy volunteers, as approved by the ethics committee of the University Medical Center of the Georg-August-University of Göttingen. To generate human induced pluripotent stem cells (hiPSCs) from primary fibroblasts derived from these skin biopsies, the lentiviral STEMCCA system was used as previously described (Streckfuss-Bömeke et al., 2013). Briefly, 1.5 × 10^4^ human primary fibroblasts were plated onto a well of a 12-well plate 1 day prior to the transduction. The viruses were produced in-house in HEK cells and the supernatant of virus productions were used. The cells were transduced in fibroblast growth medium composed of DMEM (Invitrogen) supplemented with 10% heat-inactivated fetal calf serum (FCS, Lonza), L-glutamine (2 mM, Invitrogen), 1× non-essential amino acids (NEAA, Invitrogen), β-mercaptoethanol (β-ME, 50 μM, Serva), and penicillin (50 U/mL)/streptomycin (50 μg/mL) at 37°C with 5% CO_2_ atmosphere. Additionally, polybrene (2 μg/mL, Sigma-Aldrich) was applied to the medium. After 24 h at 37°C, the virus particles were removed by washing the cells three times with DMEM and the cells were cultured in fibroblast growth medium for another 5 days. The cells were then transferred onto 6-cm cell culture dishes pre-coated with mitomysin C-inactivated mouse embryonic fibroblasts (MEFs) at a ratio of 1:4 and the medium was changed to hES-medium composed of DMEM/F12 (Invitrogen) containing 20% Knockout serum replacement (Invitrogen), L-glutamine (2 mM), 1× NEAA, β-ME (50 μM), and basic fibroblast growth factor (bFGF, 10 ng/mL, TEBU). The colonies with typical morphology for human pluripotent stem cells first appeared 4–6 weeks later and were mechanically isolated. The isolated hiPSC colonies were then expanded on MEFs in hES-medium.

#### Culture of hiPSCs and differentiation to cardiomyocytes

Adherent cultures of hiPSCs were adapted to feeder-free conditions of growth in matrigel-coated dishes, in presence of chemically defined medium (mTeSR, StemCell Technologies). Differentiation of hiPSCs to CMs was performed by following the small molecules-mediated canonical Wnt pathway modulation exactly as previously described (Lian et al., 2013). Briefly, undifferentiated adherent cultures were treated with CHIR99021 (12 μM, Selleckchem) for 24 h on day 1, followed by Wnt pathway inhibition by IWP4 (5 μM, Stemgent) on day 4 and 5 of differentiation. After culturing for 7 days in RPMI medium with B27 (Invitrogen/Life Technologies), insulin was added and differentiating cells were cultured until day 30. Enrichment for CMs was performed by adding 4 mM lactic acid in substitution of glucose (Tohyama et al., 2013) for 6 days.

#### Dissociation of cardiomyocytes

Single hiPSC-CMs were obtained by enzymatic dissociation following a protocol somewhat similar to one previously described for the isolation of rabbit sinoatrial nodal cells (Verkerk et al., 2009). The cardiomyocyte-enriched cultures were transferred manually to a low-Ca^2+^ Tyrode solution, containing (in mM): 140 NaCl, 5.4 KCl, 0.01 CaCl_2_, 1.0 MgCl_2_, 5.5 glucose, 5.0 HEPES, and 14.1 creatine; pH 7.4 (NaOH). After an incubation of 10 min at room temperature, Liberase (0.04 mg/mL, Roche Chemicals) and Elastase (0.01 mg/mL, Serva) were added and cells were incubated for 10 min at 37°C while shaking gently. Subsequently, supernatant was removed and a Kraft–Brühe solution (37°C) was added to stop the enzymatic dissociation. This Kraft–Brühe solution contained (in mM): 85 KCl, 30 K_2_HPO_4_, 5.0 MgSO_4_, 5.5 glucose, 5.0 pyruvic acid, 5.0 creatine, 30 taurine, 5.0 Na-hydroxybutyric acid, 5.0 succinic acid, 2.0 Na_2_ATP and 1% BSA; pH 7.2 (KOH). Dissociation to single cells was achieved by a short period of firm manual shaking, after which the cell suspensions were incubated at 37°C for 10 min while shaking. hiPSC-CMs were centrifuged and resuspended in basic differentiation medium, i.e. RPMI medium supplemented with B27, to which 20% FBS and penicillin (50 U/mL)/streptomycin (50 μg/mL) were added, and plated on 0.1% gelatin-coated glass coverslips. Medium was replaced with serum-free medium after 24 h and subsequently with antibiotic-free medium every 3–4 days. Electrophysiological analysis was performed 10–15 days after dissociation.

### Electrophysiology

#### Electrophysiological recordings

Electrophysiological recordings were made from single spontaneously active hiPSC-CMs through the amphotericin-B perforated patch clamp technique, using an Axopatch 200B amplifier (Molecular Devices, Sunnyvale, CA). Cells selected for experiments showed spontaneous beating upon visual inspection. The cell chamber was put on the stage of a Nikon Eclipse TE2000-U inverted phase-contrast microscope. Cells were superfused with the above Tyrode solution with 1.8 mM CaCl_2_ and without creatine. Low-resistance borosilicate glass pipettes (1.5–2.5 MΩ) were filled with a solution containing (in mM): 125 K-gluconate, 20 KCl, 5 NaCl, 10 HEPES, and 0.44 amphotericin-B; pH 7.2 (KOH). Series resistance was compensated by ≥80% and potentials were corrected for the estimated liquid junction potential of −15 mV.

Voltage control, data acquisition, and data analysis were accomplished using custom-written software running on an Apple Macintosh G4 computer equipped with a National Instruments NB-MIO-16 twelve-bit data acquisition board. Action potentials were low-pass filtered (5 kHz) and digitized at 4 kHz. Membrane currents were filtered and digitized at 2 and 5 kHz, respectively. Cell membrane capacitance was estimated by dividing the time constant of the decay of the capacitive transient in response to 5 mV hyperpolarizing voltage clamp steps from −40 mV by the series resistance, and amounted to 25 ± 4 pF (mean ± SEM, *n* = 9). All experiments were carried out at a temperature of 35–37°C.

We analyzed various action potential characteristics, including maximum diastolic potential (MDP), action potential amplitude (APA), maximal upstroke velocity [(dV/dt)_max_], and action potential duration at 90% repolarization (APD_90_). Action potential parameters from 10 consecutive action potentials were averaged.

#### Voltage clamp recordings of I_K1_

In a separate series of experiments, the amplitude of the intrinsic I_K1_ of hiPSC-CMs was assessed using the voltage clamp mode of the whole cell patch clamp configuration, using the aforementioned setup. As before, selected cells were beating upon visual inspection. I_K1_ was recorded as a barium sensitive current. First, 5 μM nifedipine was washed in to block Ca^2+^ channels, thus preventing contaminating effects of Ba^2+^ on calcium currents. Next, 1 mM BaCl_2_ was washed in to block inward rectifier channels (Schram et al., 2003). The barium sensitive current was determined by subtracting the current traces recorded in the presence of both nifedipine and Ba^2+^ from those recorded in the presence of nifedipine. The applied voltage clamp protocol consisted of a series of 100 ms steps from a holding potential of −80 mV to a test potential of −100 mV. I_K1_ was defined as the difference current at the end of the 100 ms step and was normalized for cell size through the membrane capacitance, which amounted to 33 ± 7 pF (mean ± SEM, *n* = 7).

#### Dynamic clamp

**Figure 1:**
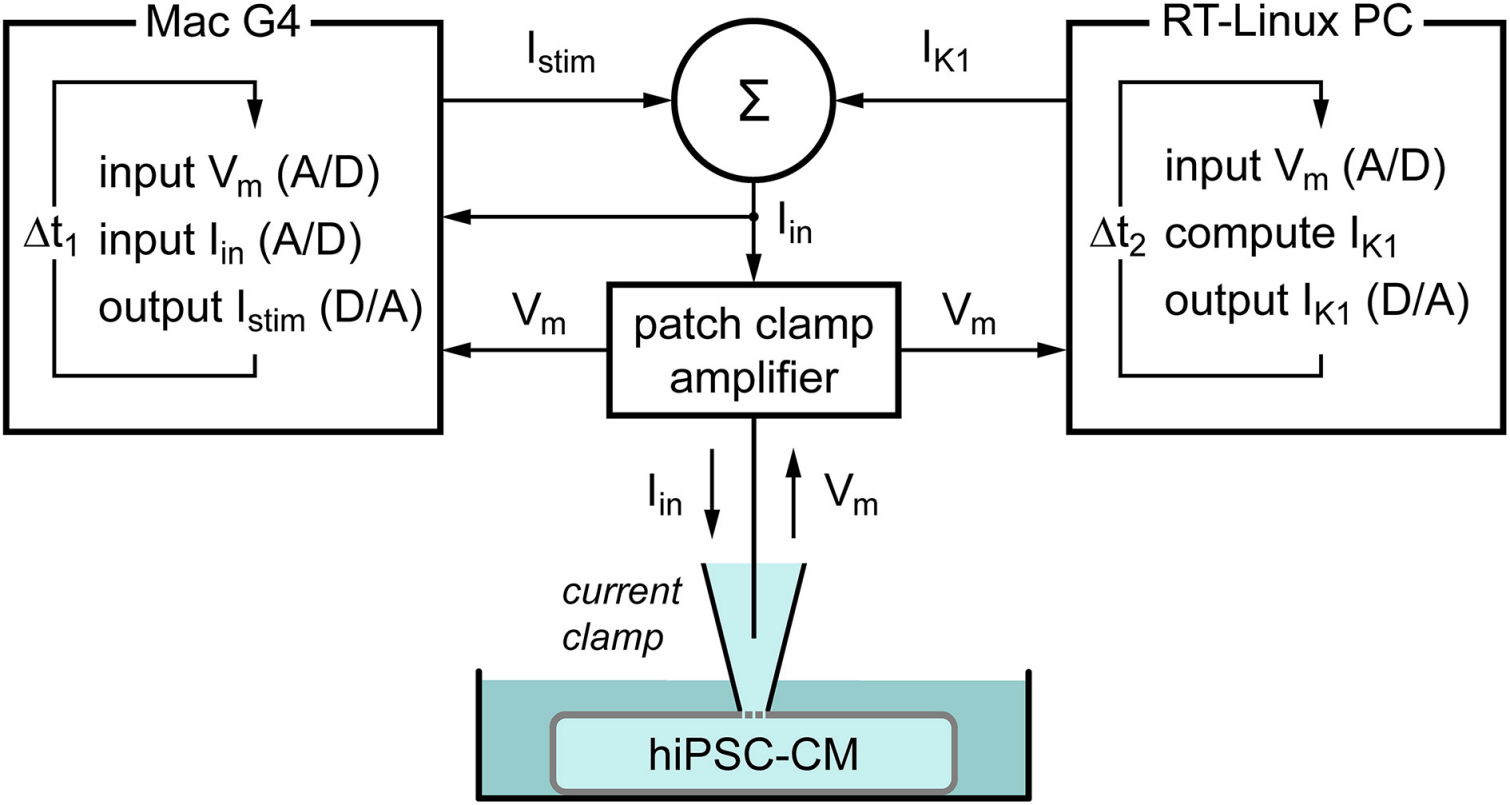
**Diagram of the experimental setup**. The membrane potential (V_m_) of a single human induced pluripotent stem cell derived cardiomyocyte (hiPSC-CM) is recorded using the perforated patch clamp technique in current clamp mode. The injected current (I_in_) is the sum of a stimulus current (I_stim_) and a virtual inward rectifier potassium current (I_K1_), which is computed in real time, based on the recorded value of V_m_ (dynamic clamp). The stimulus protocol is run on an Apple Macintosh G4 computer (left), whereas a Real-Time Linux (RT-Linux) based PC is used for the continuous computation of I_K1_ (right). Sample rates are 4 and 25 kHz, respectively (Δ*t*_1_ = 0.25 ms and Δ*t*_2_ = 40 μs).

To supply a hiPSC-CM with a controllable virtual I_K1_, we used the dynamic clamp technique (Wilders, 2006). As diagrammed in Figure 1, the membrane potential V_m_ of the hiPSC-CM, which is recorded using the perforated patch clamp technique in current clamp mode, is continuously sampled into a Real-Time Linux (RT-Linux) based personal computer. The V_m_-dependent current I_K1_ is computed and a command potential generated that, combined with a command potential for any stimulus current I_stim_, is sent to the patch clamp amplifier to inject this current into the hiPSC-CM. The virtual I_K1_ is updated at a rate of 25 kHz and both V_m_ and the injected current I_in_ are stored at a rate of 4 kHz on the standard Apple Macintosh G4 laboratory computer for offline analysis using custom software.

**Figure 2:**
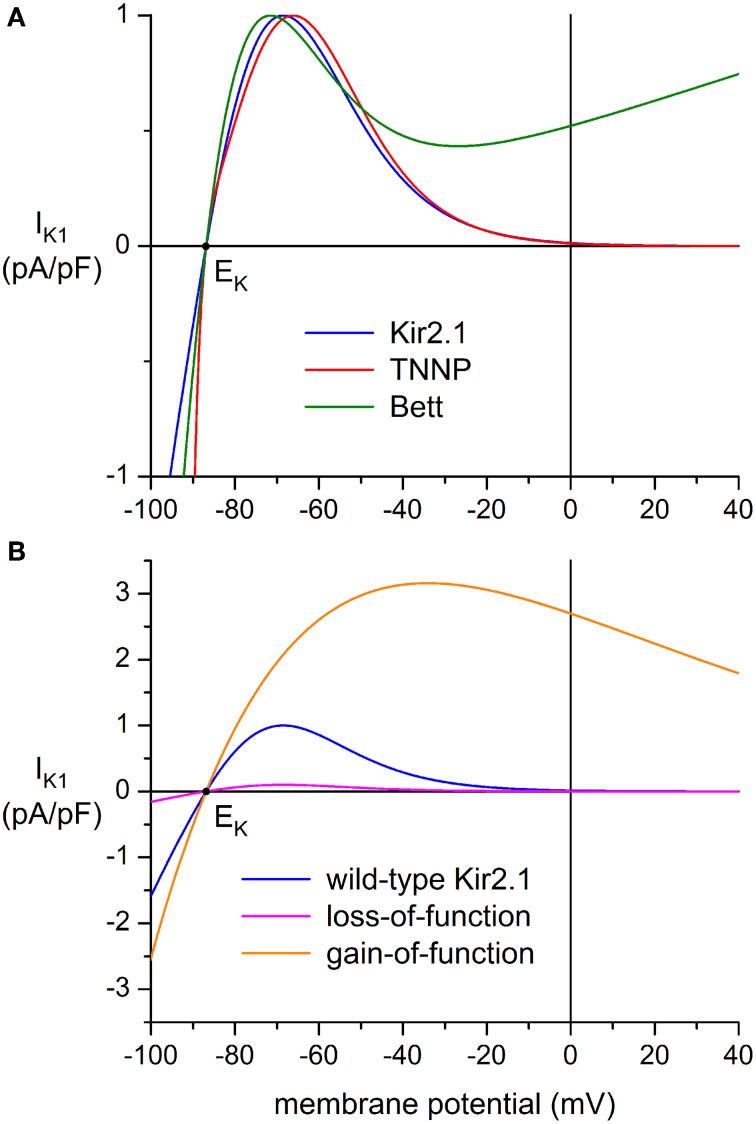
**Current-voltage relationship of inward rectifier potassium current (I_K1_) added to human induced pluripotent stem cell derived cardiomyocytes (hiPSC-CMs) through dynamic clamp. (A)** I_K1_ based on data from Kir2.1 channels expressed in HEK-293 cells by Dhamoon et al. (2004) (“Kir2.1,” blue line), I_K1_ from the human ventricular cell model by ten Tusscher et al. (2004) (“TNNP,” red line), and the synthetic I_K1_ used by Bett et al. (2013) (“Bett,” green line). Peak outward amplitude scaled to 1 pA/pF. **(B)** Current-voltage relationships for loss-of-function and gain-of-function mutations in Kir2.1, encoded by the *KCNJ2* gene. The orange line (“gain-of-function”) represents the heterozygous E299V mutation in *KCNJ2* studied by Deo et al. (2013), whereas the magenta line (“loss-of-function”) represents a dominant-negative mutation resulting in a decrease in amplitude to 10% of the wild-type Kir2.1 current of panel **(A)** (blue line). Note difference in ordinate scale between panels **(A)** and **(B)**.

Three types of wild-type (WT) I_K1_ models were used: “Kir2.1” based on a fit to data from Kir2.1 channels expressed in HEK-293 cells by Dhamoon et al. (2004), “TNNP” as used in the human ventricular cell model by ten Tusscher et al. (2004), and “Bett” as used by Bett et al. (2013) in their “electronic expression” of I_K1_ in hiPSC-CMs. The current-voltage relationships of these I_K1_ models, all scaled to a peak outward current density of 1 pA/pF, are shown in Figure 2A.

The mathematical equation for the “Kir2.1” current-voltage relationship of Figure 2A reads:

$$I_{K1}=0.12979\times \left(\frac{V_{m}\;-\;E_{K}}{1.0\;+\;e^{\left(0.093633\times \left(V_{m}+72\right)\right)}}\right)$$

In this equation, in which the rectification properties of I_K1_ are implemented through a Boltzmann equation, I_K1_ is in pA/pF and V_m_ is in mV. E_K_ is the Nernst potential for potassium, which amounts to −86.9 mV in our experimental setting.

The equation for the “TNNP” current-voltage relationship of Figure 2A reads:

$$I_{K1}\;=\;0.54419\times G_{K1}\;\times \sqrt{\frac{K_{\text{o}}}{5.4}}\times x_{K1\infty }\times (V_{m}-\;E_{K})$$

This is the equation for I_K1_ taken from the ten Tusscher et al. (2004) human ventricular cell model with a scaling factor of 0.54419 to arrive at a peak outward current density of 1 pA/pF. G_K1_ denotes the maximal I_K1_ conductance of 5.405 nS/pF and K_o_ is the extracellular K^+^ concentration of 5.4 mM, whereas x_K1∞_ is defined through:

$$x_{K1\infty }\;=\;\frac{\alpha_{K1}}{\alpha_{K1}\;+\;\beta_{K1}}$$

$$\alpha_{K1}=\;\frac{0.1}{1\;+\;e^{0.06\left(V_{m}-E_{K}-200\right)}}$$

$$\beta_{K1}=\;\frac{3e^{0.0002\left(V_{m}-E_{K}+100\right)}+e^{0.1\left(V_{m}-E_{K}-10\right)}}{1+e^{-0.5(V_{m}-E_{K})}}$$

The equation for the “Bett” current-voltage relationship of Figure 2A reads:

$$\begin{array}{l} \;I_{K1}=0.587557\times (\;0.5\;\times \left(\frac{V_{m}-\;E_{K}}{1+e^{\left(0.0896(V_{m}-\;E_{K})\right)}}\right)+\;0.01\times \\ \;\left(V_{m}-\;E_{K}\right)\;) \end{array}$$

In the original equation of Bett et al. (2013), E_K_ was set to −85 mV and a potentiometer was used to generate a standard I_K1_ amplitude of 150 pA at −75 mV, independent of cell size. Here, we used the equation of Bett et al. (2013) with the computed E_K_ of −86.9 mV and a scaling factor of 0.587557 to arrive at a peak outward current density of 1 pA/pF. In contrast with the above “Kir2.1” and “TNNP” equations, the “Bett” equation contains a linear part, which explains the shape of the current-voltage relationship at membrane potentials >−20 mV, for which the non-linear part approaches zero.

Another two I_K1_ models were used to simulate loss-of-function and gain-of-function mutations in the Kir2.1 encoding *KCNJ2* gene. A dominant-negative mutation resulting in a reduction in I_K1_ to 10% of wild-type control was used to simulate commonly observed functional effects of mutations associated with Andersen–Tawil syndrome type 1 (Nguyen et al., 2013), whereas a heterozygous gain-of-function mutation was based on the E299V mutation in *KCNJ2* that is associated with short QT syndrome type 3. Comprehensive data on this mutation were obtained by Deo et al. (2013).

The dominant-negative mutation in *KCNJ2* was simulated by scaling the above wild-type Kir2.1 based I_K1_ by a factor of 0.1, thus arriving at:

$$I_{K1}=0.012979\times \left(\frac{V_{m}-\;E_{K}}{1.0+\;e^{\left(0.093633\times \left(V_{m}\;+\;72\right)\right)}}\right)$$

This equation results in the “loss-of-function” current-voltage (I–V) relationship of Figure 2B (magenta line). The “gain-of-function” I–V relationship (Figure 2B, orange line) was obtained by fitting a “Kir2.1”-like equation to the data of Deo et al. (2013), which resulted in:

$$I_{K1}\;=\;0.98765\times \left(\frac{V_{m}-\;E_{K}}{1.0\;+\;e^{\left(0.020133\left(V_{m}\;+\;170.26\right)\right)}}\right)$$

The scaling factor of 0.98765 followed from the amplitude of the heterozygous mutant Kir2.1 current relative to wild-type Kir2.1 current reported by Deo et al. (2013).

In our dynamic clamp experiments, the hiPSC-CMs were stimulated at 1 Hz using 3-ms stimuli that ranged from 200 to 800 pA in amplitude. A virtual I_K1_, defined by any of the above equations, was added using the setup of Figure 1. The peak outward amplitude of this I_K1_ was varied between 0 and 10 pA/pF in steps of 1 or 2 pA/pF. To minimize any time effects, the order of application of I_K1_ models was varied between experiments.

### Statistics

Data are presented as mean ± SEM. Two-Way Repeated Measures ANOVA followed by the Holm–Sidak *post-hoc* test was used for comparing the different I_K1_ models and different I_K1_ amplitudes. An unpaired *t*-test with Bonferroni correction for multiple comparisons was used to compare action potential parameters of hiPSC-CMs and human ventricular myocytes. Statistical analysis was carried out with SigmaStat 3.5 software. *P* < 0.05 was considered statistically significant.

## Results

### Characteristics of hiPSC-CMs

**Figure 3:**
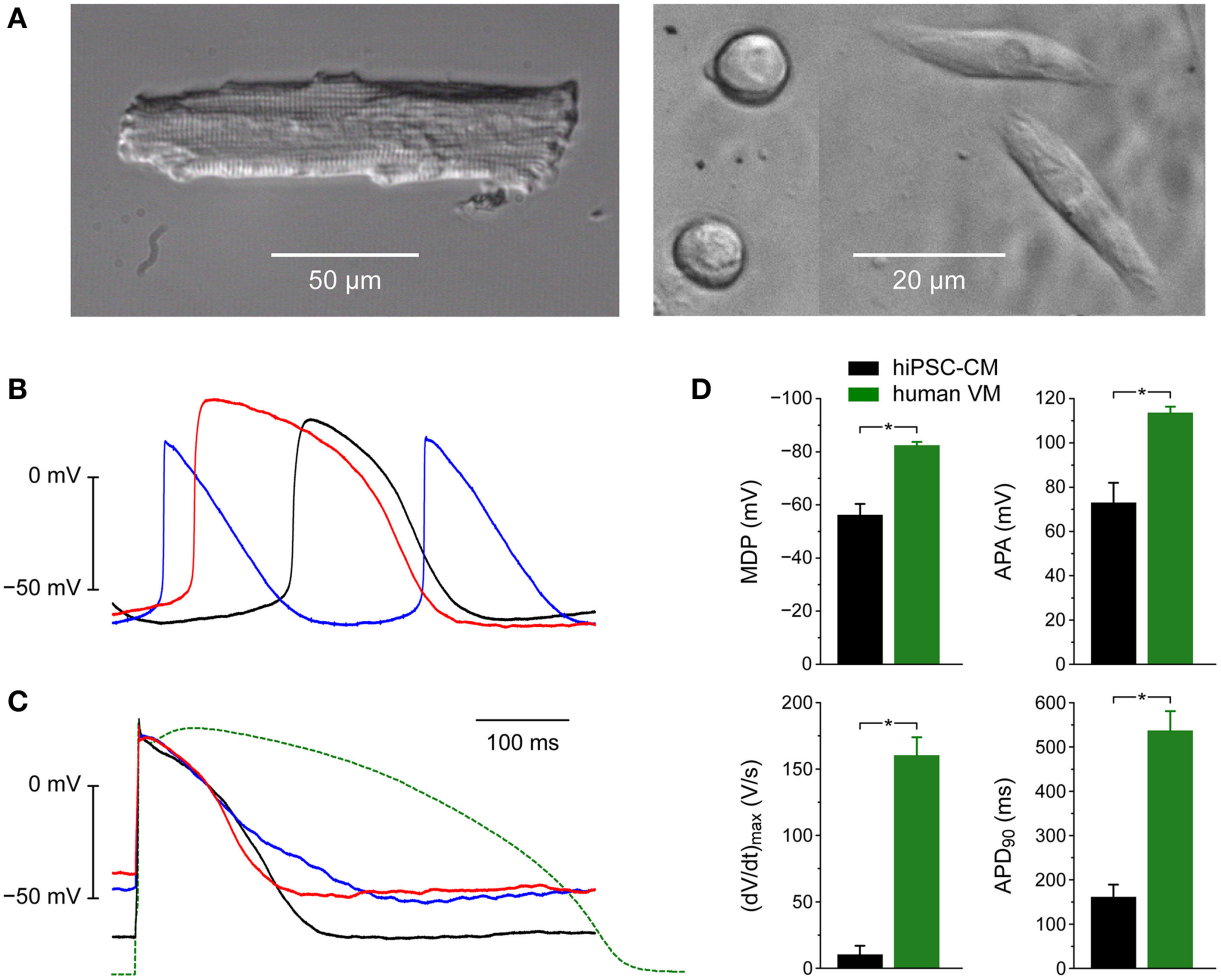
**Morphological and electrophysiological phenotype of human induced pluripotent stem cell derived cardiomyocytes (hiPSC-CMs) and native human ventricular myocytes (VMs). (A)** Phase-contrast micrographs of a typical human VM (left) and four hiPSC-CMs (right). Differently shaped hiPSC-CMs were present at close distance in the same microscope field. **(B)** Action potentials of three different spontaneously active hiPSC-CMs. **(C)** Action potentials of three different intrinsically quiescent hiPSC-CMs upon 1 Hz stimulation (solid lines) and a typical action potential of a single human VM isolated from a failing heart upon 1 Hz stimulation (dashed line). **(D)** Maximum diastolic potential (MDP), maximum upstroke velocity [(dV/dt)_max_], action potential amplitude (APA) and action potential duration at 90% repolarization (APD_90_) of 9 hiPSC-CMs (left bars) and 9 human VMs (right bars), all stimulated at 1 Hz. Human VMs were isolated from explanted hearts of male patients in end-stage heart failure (Verkerk et al., 2005), ^*^P < 0.05.

Figure 3 illustrates the morphological and electrophysiological phenotype of our hiPSC-CMs in comparison with that of native human ventricular myocytes (VMs). For these human VMs we used data that we obtained in the course of a previous study (Verkerk et al., 2005).

Whereas native human VMs are elongated and show a clear and regular cross-striated pattern (Figure 3A, left), the hiPSC-CMs are relatively small and less well-organized (Figure 3A, right). In the same dish, their appearance can be circular, with a diameter of ≈10 μm, as well as somewhat elongated.

When we made current clamp recordings from our hiPSC-CMs using the perforated patch clamp technique, we obtained widely different action potentials (Figures 3B,C). Some cells were visually beating and showed spontaneous electrical activity (Figure 3B). Others were intrinsically quiescent and action potentials could be elicited with 3 ms rectangular stimulus pulses of typically 400–600 pA. With a value of ≈−50 mV their resting membrane potential was clearly depolarized compared to that of freshly isolated native VMs (Figure 3C, solid lines vs. dashed line). Also, the action potential duration was considerably shorter, even if we take into consideration that our native VMs originate from explanted hearts of patients with end-stage heart failure and thus most likely show a prolonged action potential due to downregulation of repolarizing currents (Tomaselli and Marbán, 1999), including I_K1_ (Beuckelmann et al., 1993).

Figure 3D shows action potential parameters of 9 spontaneously beating hiPSC-CMs (left black bars) as well as 9 native human VMs (right green bars). As already apparent from Figures 3B,C the action potential amplitude (APA) of 73 ± 9 mV of the hiPSC-CMs is smaller than that of native human VMs and the action potential duration at 90% repolarization (APD_90_) of 162 ± 27 ms is shorter (Figure 3D, right). Also, the (dV/dt)_max_ of 10 ± 7 V/s is much smaller, but this may, at least partly, be due to the depolarized MDP of −56 ± 4 mV (Figure 3D, left), because such depolarization may result in almost totally inactivated fast sodium channels and thereby in a strongly reduced (dV/dt)_max_ (Berecki et al., 2010).

### Amplitude of native I_K1_

**Figure 4:**
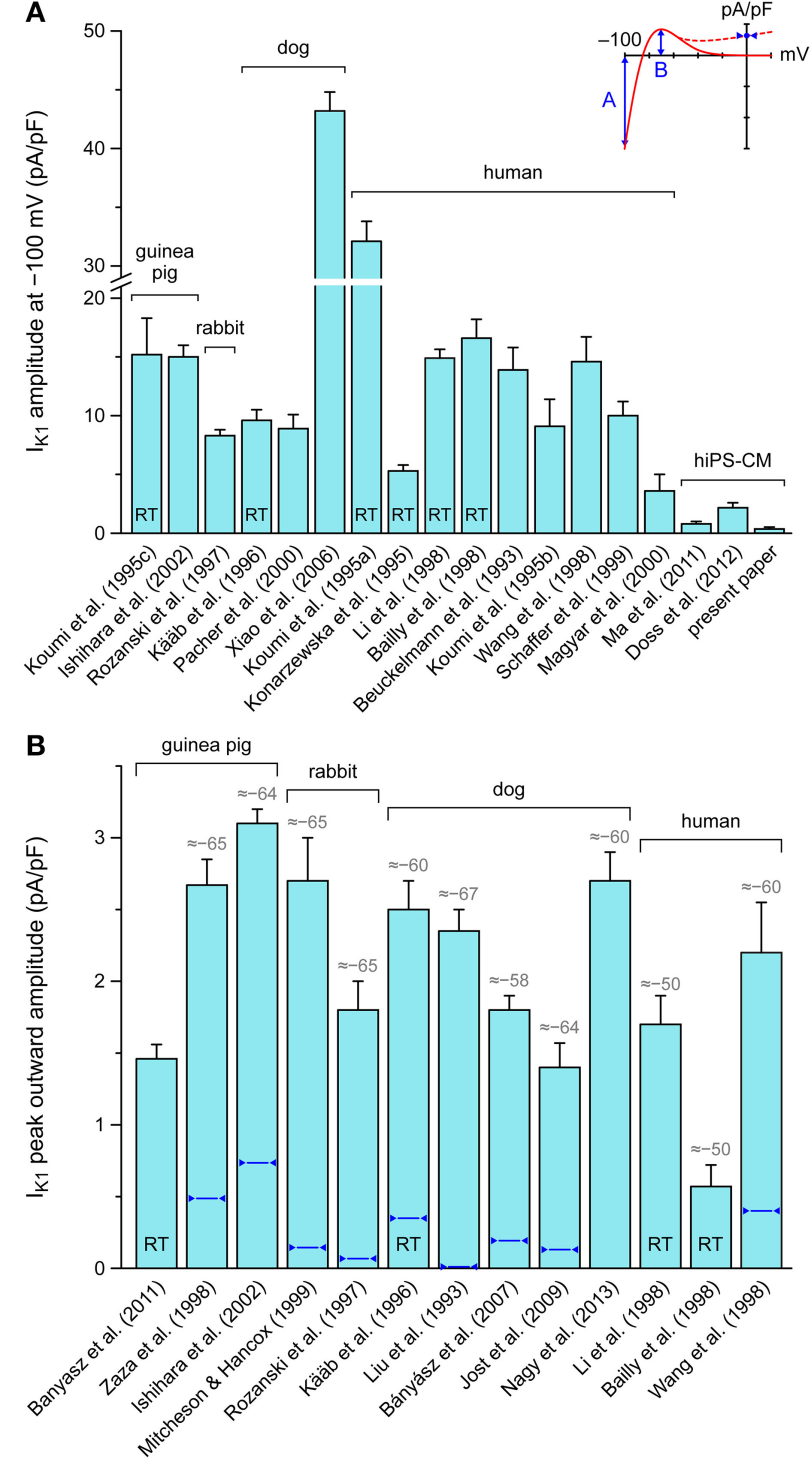
**Amplitude of inward rectifier potassium current (I_K1_) in mammalian ventricular myocytes and human induced pluripotent stem cell derived cardiomyocytes (hiPSC-CMs). (A)** Amplitude of I_K1_ at a membrane potential of −100 mV. Note axis break. **(B)** Amplitude of peak outward I_K1_. Inset illustrates current-voltage relationship of I_K1_ with values shown in panels A and B. Data are mean ± SEM obtained at room temperature (bars labeled “RT”) or at physiological or close-to-physiological temperature. If data are available, numbers in gray above bars in **(B)** indicate the membrane potential (in mV) at which the peak outward amplitude is reached, whereas dark blue triangles indicate the approximate amplitude at 0 mV. Some data are estimated from graphs.

Because we hypothesized that the depolarized MDP of our hiPSC-CMs was caused by a lack of I_K1_, we carried out voltage clamp experiments to determine the amplitude of I_K1_. In 5 out of 7 cells studied, a detectable I_K1_ was absent. The remaining two cells showed a small I_K1_ with an amplitude of 0.36 ± 0.14 pA/pF at −100 mV. To allow for a comparison with the amplitude of the native I_K1_ in isolated mammalian VMs, we carried out a literature search. Results of this search are presented in Figure 4. Results of this search are presented in Figure 4, based on data of Beuckelmann et al. (1993), Liu et al. (1993), Konarzewska et al. (1995), Koumi et al. (1995a,b,c), Kääb et al. (1996), Rozanski et al. (1997), Bailly et al. (1998), Li et al. (1998), Wang et al. (1998), Zaza et al. (1998), Mitcheson and Hancox (1999), Schaffer et al. (1999), Magyar et al. (2000), Pacher et al. (2000), Ishihara et al. (2002), Xiao et al. (2006), Bányász et al. (2007), Jost et al. (2009), Banyasz et al. (2011), Ma et al. (2011), Doss et al. (2012), and Nagy et al. (2013).

According to data from literature, there are large variations in the amplitude of I_K1_. If measured at −100 mV, this amplitude is typically ≈10 pA/pF, more or less independent of the considered species (guinea pig, rabbit, dog and human) (Figure 4A). However, canine data vary between 8.9 ± 1.2 and 43.2 ± 1.6 pA/pF (*n* = 5 and *n* = 43, respectively) and human data between 3.6 ± 1.4 and 32.1 ± 1.7 pA/pF (*n* = 4 and *n* = 14, respectively). Nevertheless, with the above 0.36 ± 0.14 pA/pF and values of 0.8 ± 0.2 (*n* = 6; Ma et al., 2011) and 2.2 ± 0.4 pA/pF (*n* = 38; Doss et al., 2012), the amplitude in hiPSC-CMs is considerably smaller than generally observed in human VMs.

In terms of functional relevance, the amplitude of the peak outward I_K1_ (labeled “B” in Figure 4A, inset) is more relevant than that at −100 mV, which is outside the physiological membrane potential range. However, data on this peak outward current are relatively scarce. As shown in Figure 4B, this peak outward amplitude is typically reached at ≈−65 mV and amounts to ≈2 pA/pF, again more or less independent of species. Human data vary between 0.57 ± 0.15 (*n* = 8; Bailly et al., 1998) and 2.2 ± 0.35 pA/pF (*n* = 6; Wang et al., 1998). Data on peak outward I_K1_ in hiPSC-CMs are limited to a peak value of 1.0 ± 0.2 pA/pF (*n* = 6; Ma et al., 2011), which was observed at a membrane potential of −35 mV rather than ≈−65 mV and is not shown in Figure 4B.

Another discrepancy between studies on I_K1_ in mammalian VMs is in the shape of the I-V relationship. In some studies, it is reported that I_K1_ approaches zero at potentials >−20 mV, whereas a substantial current is reported in others, as illustrated by the solid and dashed lines in the inset to Figure 4A. In Figure 4B, the amplitude of I_K1_ at 0 mV is indicated by dark blue triangles. It varies between 0 and 25% of the peak outward amplitude. In the “Kir2.1,” “TNNP,” and “Bett” models for I_K1_, this is 1, 1, and 52%, respectively (see Figure 2A).

### Addition of I_K1_ through dynamic clamp

Knowing that the native I_K1_ of our hiPSC-CMs is very small in comparison with that in human VMs (Figure 4), we decided to supply our hiPSC-CMs with an “I_K1_ boost” through dynamic clamp, injecting a current through the patch clamp pipette with the functional characteristics of the lacking I_K1_, as set out in Materials and Methods Section Dynamic Clamp and diagrammed in Figure 1. Given the uncertainty regarding the “normal” amplitude of I_K1_ in human VMs as well as the shape of its I–V relationship, we selected three different models of I_K1_ and varied the amplitude over a wide range.

We injected an *in silico* I_K1_ into nine different hiPSC-CMs and assessed the effects on the action potential. For each hiPSC-CM, we used each of the “Kir2.1,” “TNNP,” and “Bett” models for I_K1_, with characteristics detailed in Materials and Methods Section Dynamic Clamp and illustrated in Figure 2. Of note, the I–V relationship of the Kir2.1 and TNNP models is similar to that shown as a solid line in the inset to Figure 4A. In this respect, the Bett model is different, with a substantial current in the membrane potential range of the action potential plateau (Figure 2).

The amplitude of the injected I_K1_ was scaled according to its peak outward current density, which was set to values of 0 (no I_K1_ injected), 1, 2, 4, 6, 8, or 10 pA/pF. Thus, the three different I_K1_ models could be readily compared in the physiological membrane potential range. It should be noted, however, that an identical peak outward current density does not imply an identical amplitude at −100 mV. If, for example, the peak outward current density was set 2 pA/pF, this I_K1_ amplitude at −100 mV amounted to 3.2, 73.7, and 6.0 pA/pF for the Kir2.1, TNNP, and Bett models, respectively.

#### Injection of “Kir2.1” I_K1_

**Figure 5:**
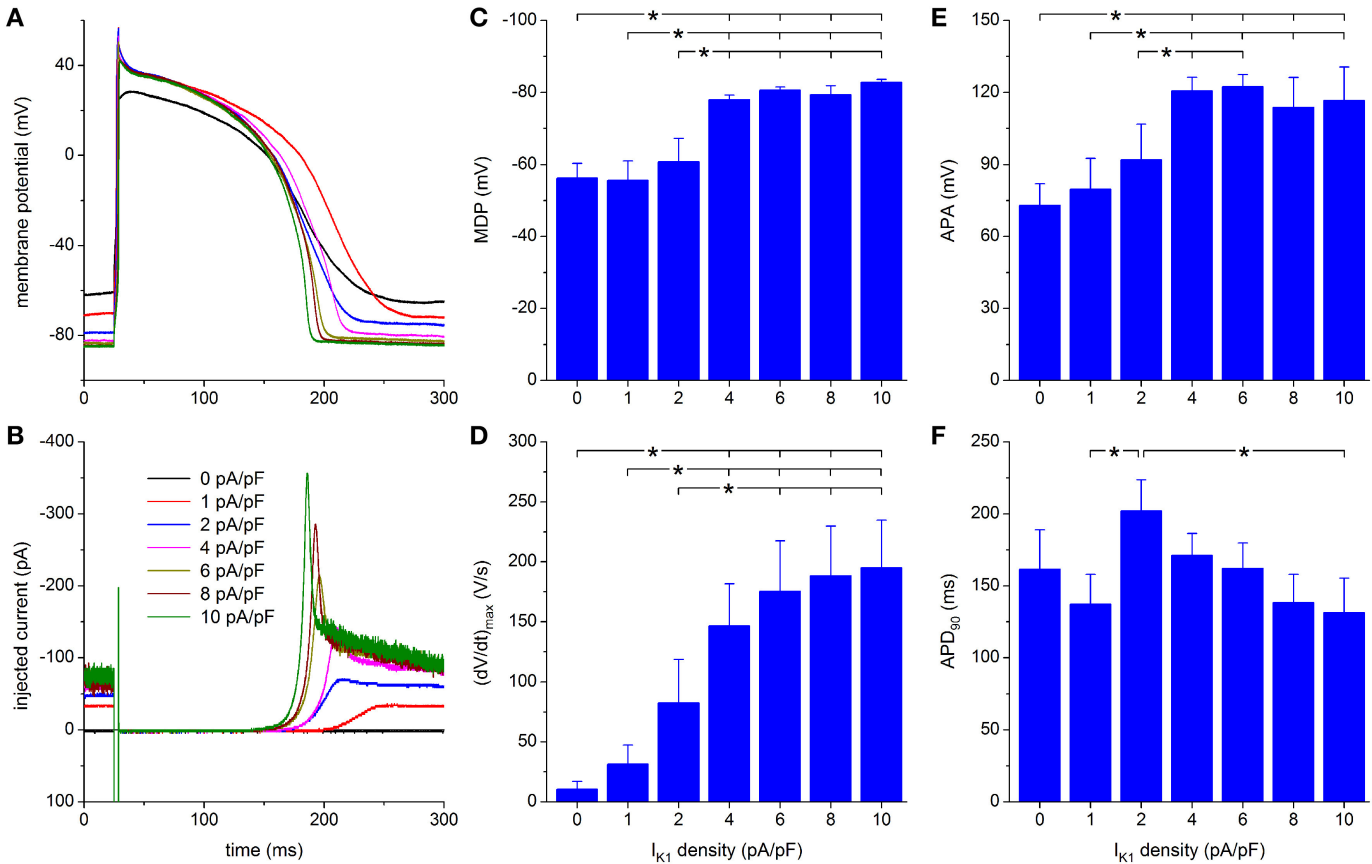
**Effect of Kir2.1-based I_K1_ on the action potential of hiPSC-CMs. (A)** Action potential of a hiPSC-CM upon injection of simulated I_K1_, which is computed in real time according to the “Kir2.1” current-voltage relationship of Figure 2A with its peak outward amplitude scaled to 0–10 pA/pF, as indicated. **(B)** Corresponding dynamic clamp current injected into the cell. The sharp peak at time 25 ms is due to the stimulus current of 3 ms duration and 600 pA amplitude. **(C–F)** Maximum diastolic potential (MDP), maximum upstroke velocity [(dV/dt)_max_], action potential amplitude (APA) and action potential duration at 90% repolarization (APD_90_) of 9 hiPSC-CMs at I_K1_ peak outward amplitudes of 0–10 pA/pF, ^*^P < 0.05.

Figure 5A shows the native action potential of a hiPSC-CM stimulated at a frequency of 1 Hz (black trace) and its action potential upon injection of a Kir2.1 I_K1_ with a peak outward density of 1–10 pA/pF. The corresponding injected current is shown in Figure 5B. With an I_K1_ of 1 pA/pF peak amplitude, the membrane potential becomes hyperpolarized by ≈7 to −72 mV, but the spontaneous diastolic depolarization of the cell is not fully suppressed. Doubling the peak amplitude of the injected I_K1_ to 2 pA/pF results in a stable RMP of −79 mV (Figure 5A, blue trace). At this potential, the injected current is ≈−50 pA (Figure 5B, blue trace), which has the functional effect of an outward, positive membrane current of 50 pA. Accordingly, the negative injected current is depicted as an upward deflection.

Upon further increasing the peak outward density of I_K1_ to 4–10 pA/pF, the RMP becomes more negative, reaching an almost steady value near −84 mV, at which the simulated I_K1_ amounts to ≈70 pA (Figures 5A,B). The increase in peak current (Figure 5B) causes a more pronounced final repolarization phase of the action potential as well as a shortening in action potential duration (Figure 5A). Also, both (dV/dt)_max_ and APA tend to increase, but this is not immediately apparent from Figure 5A.

A statistically significant difference with respect to control (zero I_K1_ addition) is found for the MDP, (dV/dt)_max_ and APA starting at a peak outward I_K1_ amplitude of 4 pA/pF (Figures 5C–E). Significant differences in MDP, (dV/dt)_max_ and APA are also found between 1 or 2 pA/pF I_K1_ and higher amplitudes (Figures 5C–E). Injection of I_K1_ with a peak outward density of 4 pA/pF results in an average RMP of −78.0 ± 1.3 mV. For the same I_K1_ amplitude, a value of 146 ± 35 V/s is found for (dV/dt)_max_. In line with Figure 5A, Figures 5C,D may suggest a further hyperpolarization of the MDP and an increase in (dV/dt)_max_ with increasing peak outward density of I_K1_, but differences were not statistically significant. Figure 5F shows a negative trend in APD_90_, from 202 ± 22 ms at 2 pA/pF to 131 ± 24 ms at 10 pA/pF, but only the APD_90_ values at 2 and 10 pA/pF are statistically significantly different.

#### Injection of “TNNP” I_K1_

**Figure 6:**
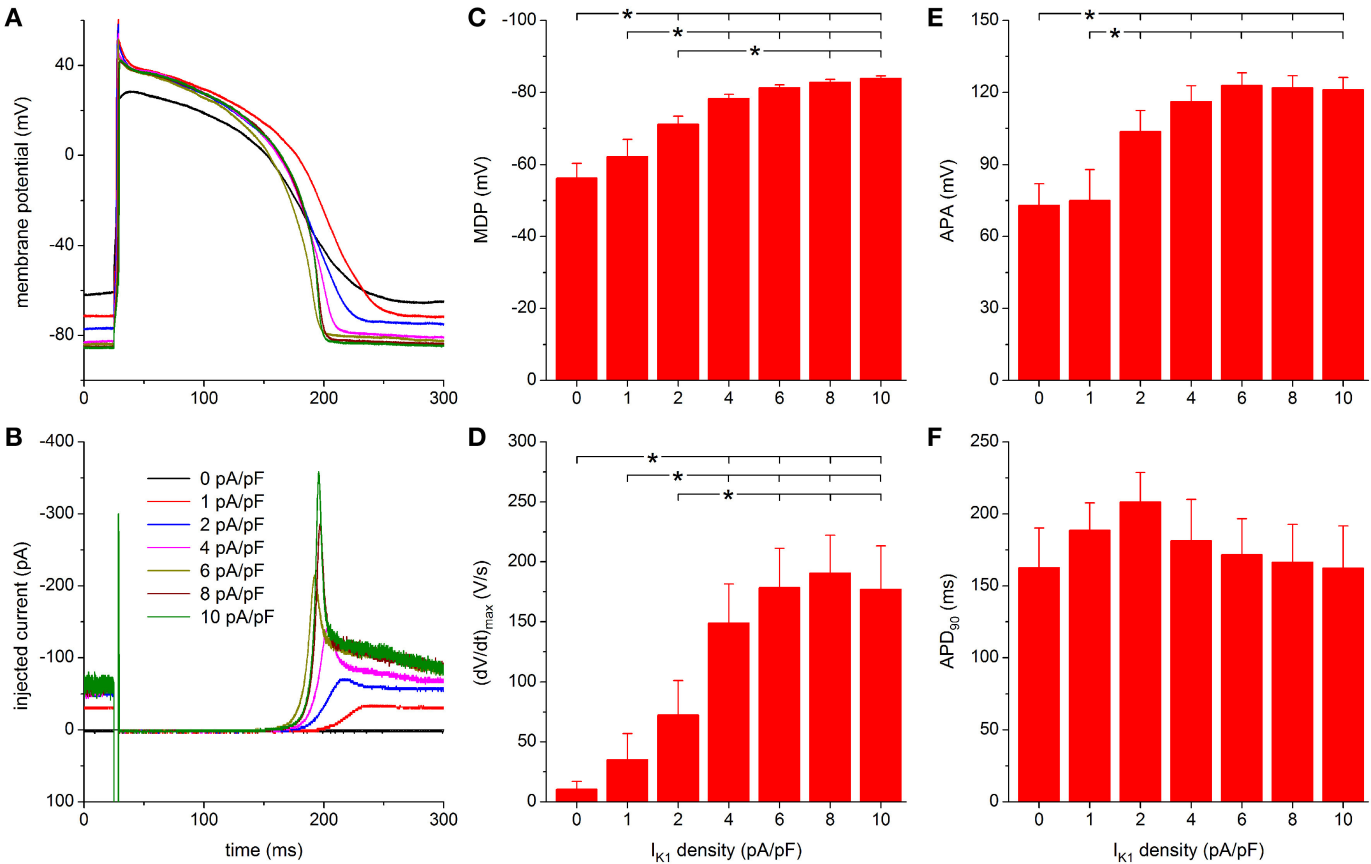
**Effect of TNNP-based I_K1_ on the action potential of hiPSC-CMs. (A)** Action potential of a hiPSC-CM upon injection of simulated I_K1_, which is computed in real time according to the “TNNP” current-voltage relationship of Figure 2A with its peak outward amplitude scaled to 0–10 pA/pF, as indicated. **(B)** Corresponding dynamic clamp current injected into the cell. The sharp peak at time 25 ms is due to the stimulus current of 3 ms duration and 600 pA amplitude. **(C–F)** Maximum diastolic potential (MDP), maximum upstroke velocity [(dV/dt)_max_], action potential amplitude (APA) and action potential duration at 90% repolarization (APD_90_) of 9 hiPSC-CMs at I_K1_ peak outward amplitudes of 0–10 pA/pF, ^*^P < 0.05.

Highly similar results were obtained with the TNNP model of I_K1_, which comes from the widely used ten Tusscher et al. (2004) model of a human ventricular cell. The outward peak is reached at a slightly less negative membrane potential than with the Kir2.1 model (−66.1 vs. −68.5 mV; Figure 2), but otherwise the I–V relationships are largely comparable within the physiological membrane potential range, positive to E_K_. Figure 6A shows the effects of injection of the TNNP I_K1_ on the action potential of the same hiPSC-CM as used for Figure 5A. The corresponding injected current is shown in Figure 6B. With an I_K1_ of 1 pA/pF peak amplitude, the membrane potential now becomes hyperpolarized by ≈6 to −71 mV. Again, the spontaneous diastolic depolarization is not fully suppressed. A stable RMP of −77 mV is achieved upon doubling the peak amplitude of the injected I_K1_ to 2 pA/pF (Figure 6A, blue trace). One may speculate that the slightly smaller hyperpolarization than with the Kir2.1 I_K1_ (−71 vs. −72 mV and −77 vs. −79 mV) reflects the slightly smaller amplitude of the TNNP I_K1_ in this membrane potential range (Figure 2A).

Further observations are highly similar to those with the Kir2.1 I_K1_ model. A stable RMP near −84 mV is reached upon further increasing the peak outward density of I_K1_ to 4–10 pA/pF and the increase in peak current causes a more pronounced final repolarization phase of the action potential as well as a shortening in action potential duration (Figures 6A,B). Also, an increase in (dV/dt)_max_ and APA is observed.

For both MDP and APA, a statistically significant difference with respect to control (zero I_K1_ addition) occurs at peak outward I_K1_ amplitudes of 2 pA/pF and higher (Figures 6C,E). For (dV/dt)_max_, statistical significance is reached at 4 pA/pF and higher (Figure 6D). Significant differences in MDP, (dV/dt)_max_ and APA are also found between 1 or 2 pA/pF I_K1_ and higher amplitudes (Figures 6C–E). Injection of I_K1_ with a peak outward density of 4 pA/pF results in an average RMP of −78.3 ± 1.2 mV. For the same I_K1_ amplitude, a value of 149 ± 33 V/s is found for (dV/dt)_max_. More hyperpolarized values for MDP and higher values for (dV/dt)_max_ are observed at 6–10 pA/pF, but differences are not statistically significant. As with the Kir2.1 I_K1_, a negative trend in APD_90_ is observed (Figure 6F), from 208 ± 20 ms at 2 pA/pF to 162 ± 29 ms at 10 pA/pF, but it is less pronounced and no statistically significant differences are obtained.

#### Injection of “Bett” I_K1_

**Figure 7:**
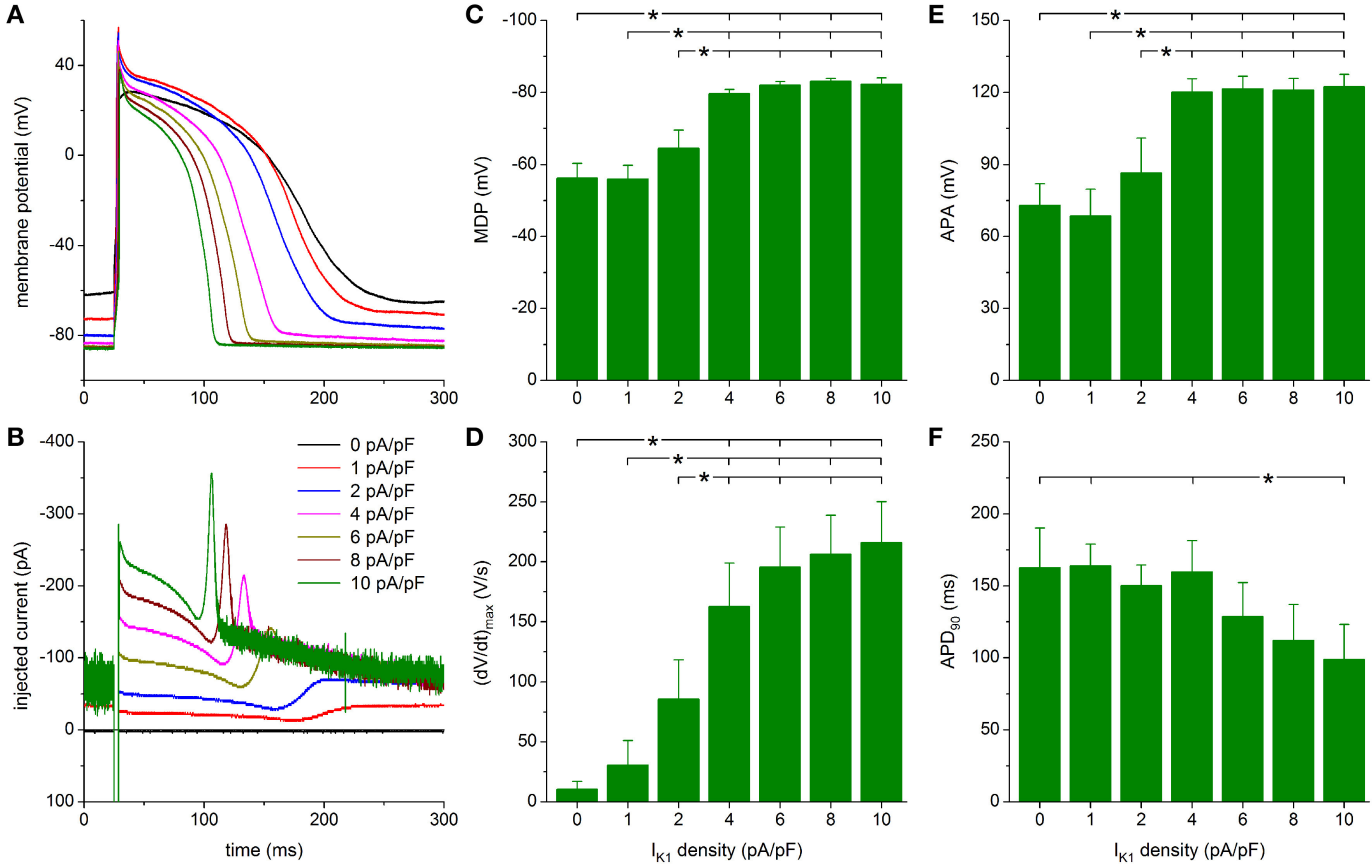
**Effect of Bett-based I_K1_ on the action potential of hiPSC-CMs. (A)** Action potential of a hiPSC-CM upon injection of simulated I_K1_, which is computed in real time according to the “Bett” current-voltage relationship of Figure 2A with its peak outward amplitude scaled to 0–10 pA/pF, as indicated. **(B)** Corresponding dynamic clamp current injected into the cell. The sharp peak at time 25 ms is due to the stimulus current of 3 ms duration and 600 pA amplitude. **(C–F)** Maximum diastolic potential (MDP), maximum upstroke velocity [(dV/dt)_max_], action potential amplitude (APA) and action potential duration at 90% repolarization (APD_90_) of 9 hiPSC-CMs at I_K1_ peak outward amplitudes of 0–10 pA/pF, ^*^P < 0.05.

With the Bett model of I_K1_, we obtained different results. This is because of the substantial current at less negative membrane potentials, which is in contrast with the Kir2.1 and TNNP models of I_K1_ (Figure 2A). Figure 7A shows the effects of injection of Bett I_K1_ on the action potential of the same hiPSC-CM as used for Figures 5A, 6A. The corresponding injected current is shown in Figure 7B. The injected current shows a clear non-zero component directly after the stimulus current, which lasts throughout the plateau phase of the action potential and results in a relatively short action potential, due to an early start of repolarization (Figures 7A,B). With an I_K1_ of 1 pA/pF peak amplitude, the membrane potential becomes hyperpolarized to −73 mV and this time the spontaneous diastolic depolarization is completely suppressed. Upon doubling the peak amplitude of the injected I_K1_ to 2 pA/pF, a stable RMP of −80 mV is observed (Figure 7A, blue trace). The early suppression of spontaneous activity and slightly larger hyperpolarization may be attributed to the larger I_K1_ at membrane potentials negative to −70 mV than with the Kir2.1 and TNNP models of I_K1_ (Figure 2A).

As with the Kir2.1 and TNNP models of I_K1_, a statistically significant difference with respect to control (zero I_K1_ addition) is found for the MDP, (dV/dt)_max_ and APA starting at a peak outward I_K1_ density of 4 pA/pF (Figures 7C–E). Significant differences in MDP, (dV/dt)_max_ and APA are also found between 1 or 2 pA/pF I_K1_ and higher amplitudes (Figures 7C–E). Injection of I_K1_ with a peak outward density of 4 pA/pF results in an average RMP of −79.6 ± 1.2 mV and an average (dV/dt)_max_ of 163 ± 36 V/s. Slightly more hyperpolarized values for MDP and higher values for (dV/dt)_max_ are observed at 6–10 pA/pF, but differences are not statistically significant. As already apparent from Figures 7A,B, the substantial plateau I_K1_ results in pronounced action potential shortening. In particular, APD_90_ decreases from 160 ± 22 ms at 4 pA/pF to 99 ± 24 ms at 10 pA/pF. However, it should be noted that only the APD_90_ at 10 pA/pF differs statistically significantly from the APD_90_ at some of the lower I_K1_ densities (Figure 7F).

#### Comparison of I_K1_ models

**Figure 8:**
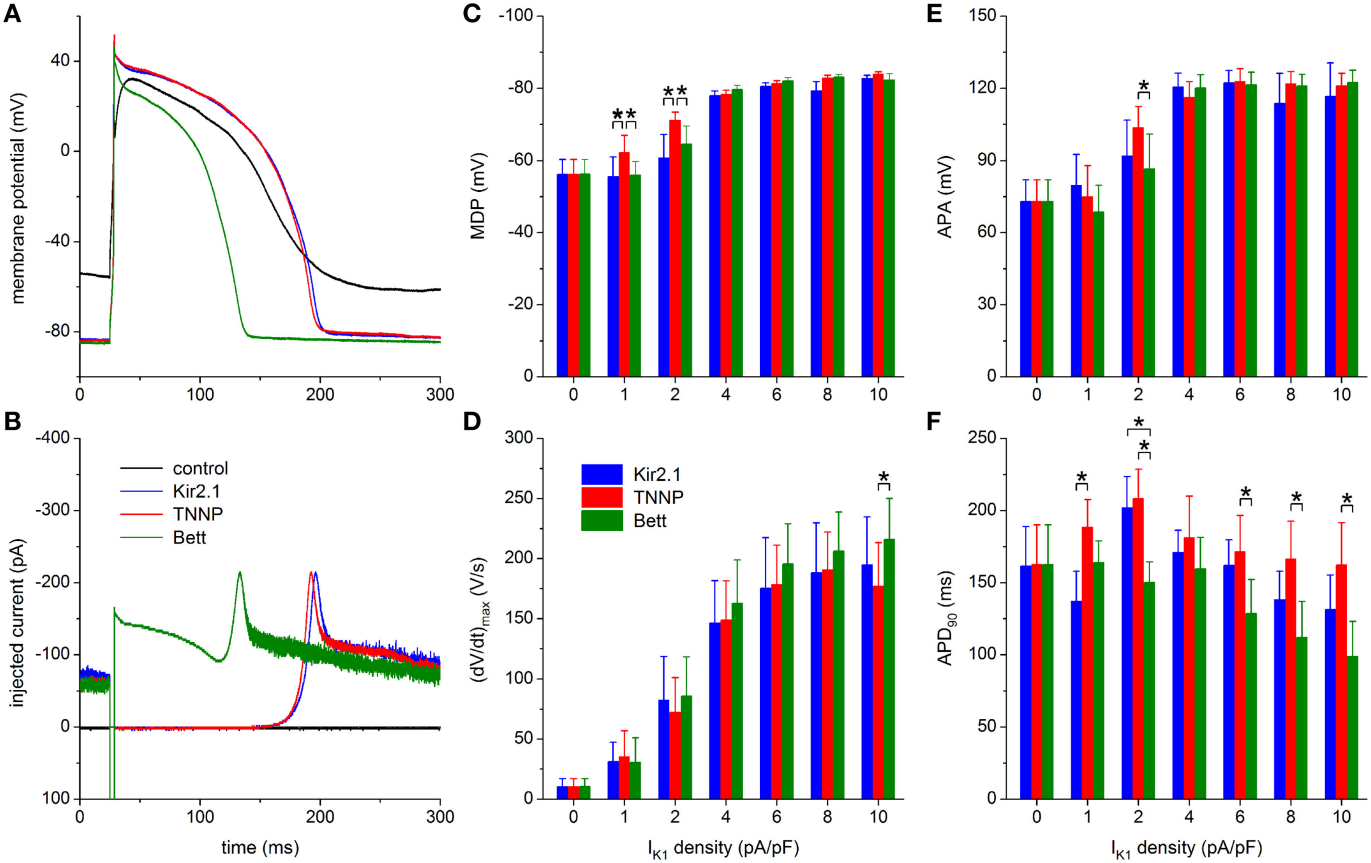
**Dynamic clamp with different I_K1_ models. (A)** Control action potential of a hiPSC-CM (black trace) and action potential of this cell upon injection of I_K1_ computed in real time according to the “Kir2.1,” “TNNP,” or “Bett” I_K1_ models of Figure 2A (blue, red, and green lines, respectively), all with a peak outward amplitude of 6 pA/pF. **(B)** Corresponding dynamic clamp current injected into the cell. The sharp peak at time 25 ms is due to the stimulus current of 3 ms duration and 600 pA amplitude. **(C–F)** Comparison of maximum diastolic potential (MDP), maximum upstroke velocity [(dV/dt)_max_], action potential amplitude (APA) and action potential duration at 90% repolarization (APD_90_) of 9 hiPSC-CMs obtained with each of the three I_K1_ models. Asterisks indicate statistically significant differences between I_K1_ models at each of the applied I_K1_ peak outward amplitudes.

In Figure 8 the results obtained with the three different models for I_K1_ are compared, thus concretizing differences between the three models. The typical effects on the action potential of the Kir2.1 and TNNP I_K1_ models, obtained with an I_K1_ peak amplitude of 6 pA/pF and another hiPSC-CM than in the typical examples of Figures 5–7, are highly similar, but the Bett I_K1_ model causes a shorter action potential (Figure 8A). This shorter action potential is in line with the injected current, which shows a substantial Bett I_K1_ during the early repolarization phase and plateau phase of the action potential (Figure 8B). The average APD_90_ at an I_K1_ peak amplitude of 6 pA/pF amounts to 129 ± 24 ms for the Bett model vs. 162 ± 18 and 171 ± 25 ms for the Kir2.1 and TNNP I_K1_ models, respectively (Figure 8F). A similar pattern is seen at other I_K1_ peak amplitudes in the 2–10 pA/pF range. Yet, statistically significant differences in APD_90_ are mainly observed between the TNNP and Bett I_K1_ models (Figure 8F).

MDP and APA values are highly similar between the three I_K1_ models, except for statistically significant differences found at the lower I_K1_ peak amplitudes of 1 and 2 pA/pF (Figures 8C,E). Both MDP and APA seem to “saturate,” at ≈−80 and ≈120 mV, respectively, for the higher amplitudes of 4–10 pA/pF. Such saturation is somewhat less apparent for (dV/dt)_max_ (Figure 8D), but it reaches typical values of 160–200 V/s for each of the models. One may argue that the statistically significant difference in (dV/dt)_max_ at 10 pA/pF reflects the shorter APD_90_ with the Bett model, which results in a longer interval for recovery of sodium channels between consecutive action potentials.

### Functional effects of I_K1_ channelopathies

**Figure 9:**
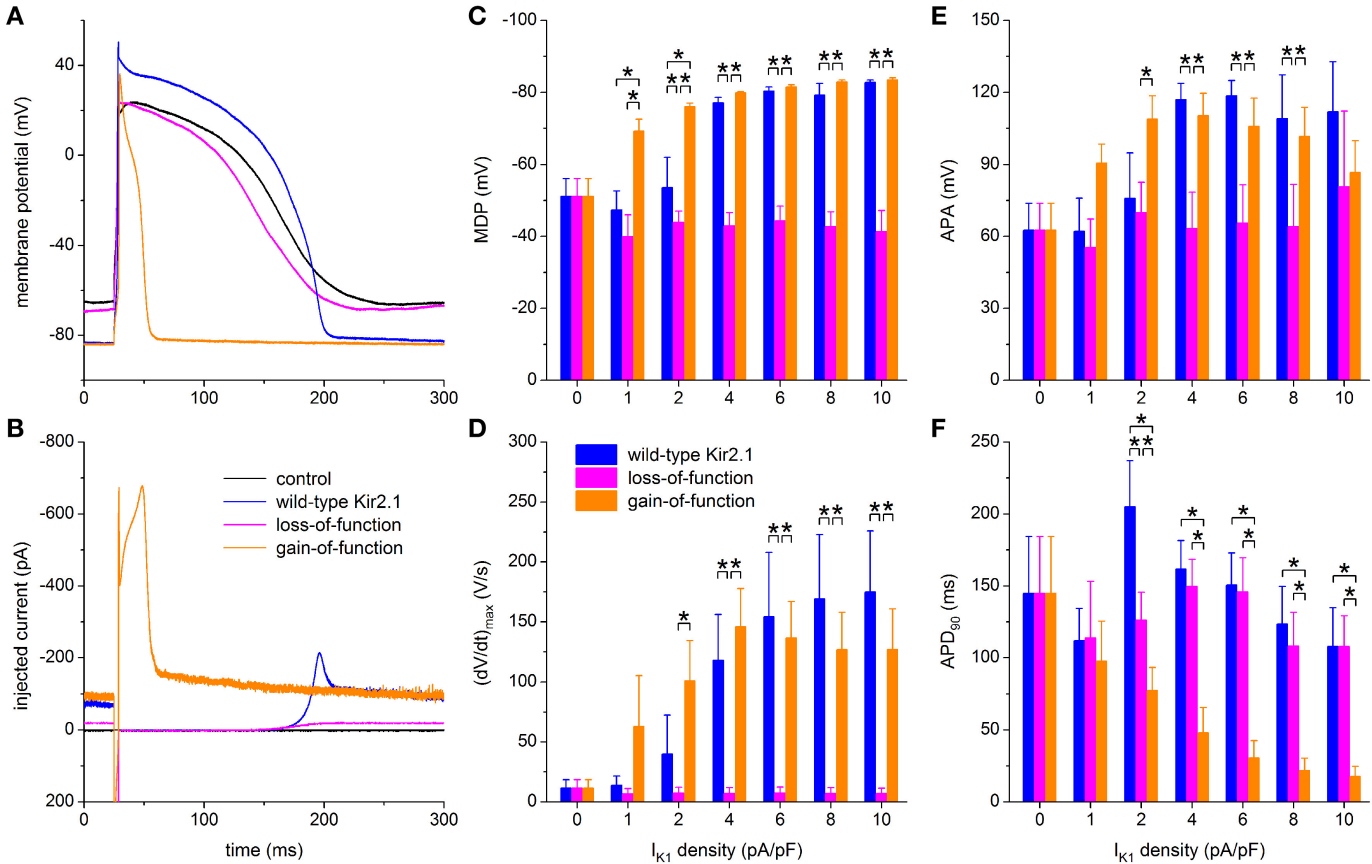
**Effect of *in silico* mutations in Kir2.1 assessed with dynamic clamp. (A)** Control action potential of a hiPSC-CM (black trace) and action potential of this cell upon injection of I_K1_ computed in real time according to the “wild-type Kir2.1,” “loss-of-function,” or “gain-of-function” I_K1_ models of Figure 2B (blue, magenta, and orange lines, respectively), all scaled by a factor of 6, thus producing a wild-type I_K1_ peak outward amplitude of 6 pA/pF. **(B)** Corresponding dynamic clamp current injected into the cell. The sharp peak at time 25 ms is due to the stimulus current of 3 ms duration and 600 pA amplitude. **(C–F)** Comparison of maximum diastolic potential (MDP), maximum upstroke velocity [(dV/dt)_max_], action potential amplitude (APA) and action potential duration at 90% repolarization (APD_90_) of 6 hiPSC-CMs obtained with each of the three I_K1_ models. Asterisks indicate statistically significant differences between I_K1_ models at each of the applied wild-type I_K1_ peak outward amplitudes.

To explore possible applications of our technique, we assessed both loss-of-function and gain-of-function mutations in Kir2.1, which is encoded by the *KCNJ2* gene and is the primary component of the ventricular I_K1_ channel. To simulate a loss-of-function mutation we reduced the wild-type Kir2.1 based I_K1_ to 10% of control, in line with the functional effects that are commonly observed in case of heterozygous dominant-negative mutations in *KCNJ2* associated with the Andersen–Tawil syndrome (type 1; ATS1). As a gain-of-function mutation we chose the heterozygous E299V mutation, which is associated with the short QT syndrome (type 3; SQT3). The corresponding I–V relationships are shown in Figure 2B, in comparison with the wild-type relationship. Experiments were carried out in a total of 6 hiPSC-CMs and the peak amplitude of the wild-type Kir2.1 was varied over the same 0–10 pA/pF as before. Figures 9A,B show a typical example at a wild-type peak amplitude of 6 pA/pF and Figures 9C–F show the average action potential parameters of the 6 hiPSC-CMs. Note that the 0–10 pA/pF I_K1_ peak amplitude values in Figures 9C–F are those for the wild-type case. The corresponding peak amplitudes are 0–1 pA/pF (10% of control) and 0–31.6 pA/pF for the loss-of-function and gain-of-function mutations, respectively, in line with the I–V relationships of Figure 2B.

Simulation of the gain-of-function mutation results in injection of an I_K1_ with such a large amplitude immediately following the action potential upstroke that the action potential is dramatically shortened (Figures 9A,B, orange traces). Such shortening is not limited to the I_K1_ density of 6 pA/pF in Figures 9A,B. A statistically significant decrease relative to wild-type Kir2.1 occurs at each of the I_K1_ densities >1 pA/pF (Figure 9F, right orange bars vs. left blue bars). No statistically significant differences vs. wild-type are observed in (dV/dt)_max_ and APA (Figures 9D,E), but RMP is more hyperpolarized, with full suppression of spontaneous depolarization, at 1 and 2 pA/pF (Figure 9C).

Although not statistically significant, the gain-of-function (dV/dt)_max_ seems to become lower than the wild-type (dV/dt)_max_ at higher I_K1_ densities (Figure 9D). At first sight, this may suggest a lower functional availability of fast sodium channels, despite the longer time for recovery from inactivation at RMP in case of the gain-of-function mutation as a result of the shortened APD_90_ (Figure 9F). However, the somewhat lowered (dV/dt)_max_ may be due to the large gain-of-function I_K1_ that flows during the upstroke (Figure 9B), especially at high I_K1_ densities, rather than a lower functional availability of fast sodium channels.

In contrast, the injection of a loss-of-function I_K1_ has only marginal effects, if any, on each of the action potential parameters, if compared to zero current injection (Figures 9C–F). Spontaneous depolarization is not remarkably suppressed throughout the range of I_K1_ densities, which is not surprising since the maximum peak outward amplitude is effectively 1 pA/pF because of the reduction to 10% of wild-type. Compared to wild-type I_K1_ with a peak density >2 pA/pF, MDP is depolarized (Figure 9C), (dV/dt)_max_ is diminished (Figure 9D) and APA is considerably reduced (Figure 9E), whereas APD_90_ is virtually unaffected (Figure 9F). Overall, the hiPSC-CM without I_K1_ injection can be regarded as a model for severe ATS1.

## Discussion

In the present study, we increased the expression level of I_K1_ in our hiPSC-CMs by adding *in silico* I_K1_ using a dynamic patch clamp approach. First, we assessed the effects of the type and magnitude of the added I_K1_. Three different models of I_K1_ were used, i.e., the “Kir2.1,” “TNNP,” and “Bett” models, and the peak outward density of the inserted I_K1_ was varied between 1 and 10 pA/pF. Next, we assessed the effects of both loss- and gain-of-function mutations in I_K1_ by modifying our *in silico* I_K1_, thus simulating ATS1 and SQT3, respectively.

### Characteristics of hiPSC-CMs

Among our hiPSC-CMs, we observed both intrinsically quiescent and spontaneously beating cells (Figures 3B,C). However, for the experiments in the present study, we selected spontaneously active hiPSC-CMs, because we expected those to exhibit a smaller I_K1_ than the quiescent ones. We have no data regarding the expression level of native I_K1_ in each individual hiPSC-CM used in our dynamic clamp experiments, but our voltage clamp experiments on the same type of cells demonstrate an almost negligible I_K1_, which is even smaller than in the hiPSC-CMs of Ma et al. (2011) and Doss et al. (2012). It should be kept in mind that the selection of spontaneously beating cells for our experiments may have led to the use of relatively immature hiPSC-CMs.

With an MDP of −56 ± 4 mV and an APA of 73 ± 9 mV (Figure 3D), our hiPSC-CMs exhibit a relatively depolarized MDP and low APA compared to those observed in other studies on hiPSC-CMs, as summarized in Table 1 of the review by Hoekstra et al. (2012). The (dV/dt)_max_ of 10 ± 7 V/s is also low, but that may reflect a low functional availability of sodium channels due to the depolarized MDP, as does the relatively low APA. Most striking, however, is the relatively short APD_90_ of 162 ± 27 ms, similar to that of 173.5 ± 12.2 ms (*n* = 16) in the hiPSC-CMs of Davis et al. (2012), who selected quiescent rather than spontaneously beating hiPSC-CMs for their action potential recordings, but much shorter than that of 955 ± 103 ms (*n* = 38) and 967 ± 141 ms (*n* = 30) in the spontaneously beating and quiescent hiPSC-CMs, respectively, of Bett et al. (2013).

We have not made specific attempts to resolve the mechanism underlying the spontaneous activity of our hiPSC-CMs. In our voltage clamp experiments, there were no signs of a functional hyperpolarization-activated “pacemaker current” or “funny current” (I_f_). This current had been observed in hiPSC-CMs by Ma et al. (2011), which are the same type of cells that Bett et al. (2013) used in their dynamic clamp study. However, a strong reduction of I_K1_ is sufficient *per se* to generate spontaneous activity in VMs, as demonstrated by suppression of I_K1_ through barium block in rabbit and guinea pig papillary muscle (Antoni and Oberdisse, 1965; Aomine, 1989) and isolated guinea pig VMs (Valenzuela and Vassalle, 1989), through viral gene transfer in guinea pig VMs (Miake et al., 2003), and in computer simulations (Doss et al., 2012; Nguyen et al., 2013).

### I_K1_ model and dynamic clamp setup

The “Kir2.1” model of Dhamoon et al. (2004) is based on the characteristics of inward rectifier current through Kir2.1 channels expressed in HEK-293 cells. The “TNNP” model stems from the mathematical model of a human ventricular myocyte by ten Tusscher et al. (2004), which is known as the TNNP model. Whereas the I–V relationships of the Kir2.1 and TNNP models are largely similar, the I–V relationship of the “Bett” model, which is based on the “electronic expression” of I_K1_ in hiPSC-CMs by Bett et al. (2013), is different, with a less pronounced rectification (Figure 2A). A common feature of the three I_K1_ models is the absence of any time dependence of I_K1_, which is in line with the virtually instantaneous kinetics of I_K1_ in the membrane potential range of the ventricular action potential (Dhamoon and Jalife, 2005). It is, however, a straightforward operation to let the dynamic clamp PC generate a time dependent I_K1_.

The setup of Figure 1, which is comparable to that of Bett et al. (2013), is somewhat complicated. In daily practice, it would be preferable to have the same computer both control the patch clamp experiment and generate the *in silico* I_K1_ rather than having two computers perform these tasks and externally summing their command potentials. In case of a time independent current like I_K1_, one may also use a dedicated patch clamp amplifier, such as the Power1401 by Cambridge Electronic Design, to realize the particular dynamic clamp configuration, which is also known as “conductance injection,” “reactive current clamp,” and “model reference current injection” (Wilders, 2006), and was designated as “real-time current simulator” by Bett et al. (2013).

### Magnitude and rectification of I_K1_—which model to use?

Data from literature are far from unequivocal regarding the magnitude and rectification of I_K1_ in mammalian VMs (see Figure 4). Differences in recording conditions can only partly explain the observed variety in experimental data. For example, data acquired at room temperature (labeled “RT” in Figure 4) rather than physiological temperature do not consistently show a smaller I_K1_. Yet, from the reported Q_10_ of 1.5 ± 0.3 (mean ± SD, *n* = 7; Kiyosue et al., 1993), one would expect an approximate doubling of the magnitude of I_K1_ upon raising the temperature from RT to physiological values. We refrained from “correcting” the data in Figure 4 for temperature, which may have resulted in an underestimation of the magnitude of I_K1_. A further, also uncorrected underestimation may result from the determination of I_K1_ as a barium dependent current (with widely different Ba^2+^ concentrations, ranging from 10 μM to 1 mM), because barium block may be incomplete at the membrane potential of peak outward I_K1_ (Bányász et al., 2007). Other issues, apart from technical limitations, that may have had impact on the data in Figure 4, are the dependence of I_K1_ magnitude on extracellular potassium concentration, which is not identical in the listed papers, and differences in corrections for liquid junction potential.

It is difficult to decide on a “physiological” value, or range of values, for the peak outward amplitude of I_K1_. Nevertheless, from the data in Figure 4B, an estimate of 2 pA/pF seems reasonable. Interestingly, this is close to its value in several human ventricular cell models (Iyer et al., 2004; ten Tusscher et al., 2004; Grandi et al., 2010). Another point of uncertainty is the degree of rectification in the I–V relationship of I_K1_ (Figure 4A, inset), although a less pronounced rectification in atrial vs. ventricular CMs is a more or less consistent finding. This variable degree of rectification, which seems attributable to different expression levels of specific Kir2.x subunits (Wang et al., 1998), is why we tested qualitatively different I–V relationships. The Kir2.1 and TNNP I–V relationships may be regarded as “ventricular-like,” whereas the I–V relationship of the Bett I_K1_ is more “atrial-like,” although it was intended by Bett et al. (2013) as an intermediate I–V relationship that could be “optimized for cell specificity.”

From the data in Figures 5–7, we conclude that an outward peak current density of 4–6 pA/pF is required to establish a stable RMP near -80 mV, associated with a (dV/dt)_max_ of 160–170 V/s and an APA of 120 mV, independent of the I_K1_ model used. Also, around 6 pA/pF, the hiPSC-CM + I_K1_ model system studied does not seem to be sensitive in its action potential parameters. Interestingly, these action potential parameter values are comparable to those of human VMs (Figure 3D), although it should be kept in mind that the data of Verkerk et al. (2005) were obtained with VMs isolated from explanted hearts of patients suffering from end-stage heart failure, which may have led to remodeling of membrane currents and thereby to changes in action potential parameters. The less pronounced rectification of the Bett I_K1_ results in typically shorter APD_90_ values than with the other two I_K1_ models (≈145 vs. ≈170 ms), as becomes apparent from Figure 8. In either case, APD_90_ is considerably shorter than that of native human VMs.

Bett et al. (2013) also noted that a stable RMP could be obtained through “electronic expression” of I_K1_. It is, however, difficult to directly compare their findings to ours, not only because of the differences in recording conditions (physiological vs. room temperature, perforated vs. whole cell patch clamp), but also because Bett et al. (2013) did not vary the magnitude of their electronically expressed I_K1_. They used the same magnitude of I_K1_ for all experiments because “this makes for simplicity in application and aspects such as quickly distinguishing between atrial and ventricular cells.” The potentiometer in their experimental setup “was set to provide a standard outward current of 150 pA at −75 mV.” With their E_K_ of −85 mV, this translates to a peak outward I_K1_ of 165 pA near −70 mV. Unfortunately, Bett et al. (2013) did not specify the membrane capacitance of their cells, but an estimate can be made because they used the commercially available iCell cardiomyocytes (Cellular Dynamics, Madison, WI). According to Ma et al. (2011), these cells show a capacitance of 35.0 ± 2.5 pF. If we assume that the hiPSC-CMs used by Bett et al. (2013) had a similar capacitance, the peak outward density of their I_K1_ amounts to ≈4.7 pA/pF (at room temperature), which is within the aforementioned range of 4–6 pA/pF required to establish a stable RMP. In a total of 31 “stimulated cells with artificial I_K1_,” Bett et al. (2013) observed an RMP of −84 ± 0.1 mV, an APA of 132 ± 2 mV, and a (dV/dt)_max_ of 147 ± 11 V/s, which is comparable to our data. However, their APD_90_, which can only be estimated from graphs, shows a wide variety with typical values around 800 ms and a maximum near 1800 ms.

### I_K1_ channelopathies

As long as hiPSC-CMs express a negligible I_K1_, it is impossible to use hiPSC-CMs derived from ATS1 or SQT3 patients as a model system to study both loss-of-function or gain-of-function mutations in *KCNJ2*. It is, however, possible to study the effects of such mutations in the setting of hiPSC-CMs through dynamic clamp as we did in our experiments that are summarized in Figure 9. In this way, we were able to demonstrate that the E299V gain-of-function mutation causes a dramatic shortening of the action potential (Figures 9A,F). This cellular observation is in line with the clinically observed exceedingly short QT interval with merging of the QRS complex and T wave (Deo et al., 2013).

As shown in Figure 9, simulation of a dominant-negative loss-of-function mutation has only little effect on the intrinsic action potential of our hiPSC-CMs. This suggests that the VMs of ATS1 patients exhibit an unstable and depolarized resting membrane potential. This would result in a reduced functional availability of fast sodium channels and thereby in a lengthening of the QRS interval. However, this is not a common observation in ATS1 patients. On the other hand, such QRS prolongation has been found in some but not all mouse models of ATS1 (Nguyen et al., 2013). Hopefully, it will become possible to improve the maturation process—thus “engineering adolescence” (Yang et al., 2014)—and generate more mature hiPSC-CMs, with a clear expression of I_K1_, that allow further research into this intriguing issue.

### Applications of virtual I_K1_

hiPSC-CMs are commonly seen as a promising model for the cardiac research field. Spontaneously active hiPSC-CMs, or—less commonly—intrinsically quiescent with a relatively depolarized RMP, are frequently used in patch clamp studies on cardiac ion channelopathies. However, the abnormal action potential profile, and associated abnormal activation of specific membrane currents, may make the hiPSC-CM an untrustworthy model for the study of certain ion channelopathies. In particular, as for example noted by Davis et al. (2012), hiPSC-CMs studies on mutations in the fast sodium channel should be interpreted with care. The aim of the present study was to investigate the feasibility of injecting the missing I_K1_ through dynamic clamp and thus make the hiPSC-CM exhibit a more native ventricular cardiomyocyte-like action potential.

Our study shows that it is feasible to let spontaneously beating hiPSC-CMs exhibit a ventricular-like action potential with a near-physiological resting membrane potential, maximum upstroke velocity and action potential amplitude through the injection of an *in silico* I_K1_ with a peak outward density of 4–6 pA/pF. The simulated degree of rectification does primarily affect the action potential duration. We suggest the use of the Kir2.1 model for I_K1_ with a peak outward density near 5 pA/pF. One may prefer the addition of a linear component—as in the Bett model, but with as substantially smaller amplitude (e.g., an amplitude at 0 mV of 10–20% of peak outward amplitude)—to simulate a less strong rectification, but this is not highly pertinent. As long as research into improving the expression of I_K1_ in hiPSC-CMs is going on, it may be helpful to improve their action potential profile by making them express a virtual I_K1_ through dynamic clamp.

In conclusion, we can state that injection of *in silico* I_K1_ makes the hiPSC-CM a more reliable model for investigating mechanisms underlying cardiac arrhythmias.

### Conflict of interest statement

The authors declare that the research was conducted in the absence of any commercial or financial relationships that could be construed as a potential conflict of interest.
